# Evaluation of Orthodontic Treatment Modalities for Obstructive Sleep Apnoea: A Systematic Review

**DOI:** 10.7759/cureus.65161

**Published:** 2024-07-22

**Authors:** Praveen Kumar Gorikapudi, Vedant Chhabria, Kirandeep Kaur, Padmanathan Ramasamy, Sherin Jeeboy, Rohini Venkatesh, Aishwarrya P.

**Affiliations:** 1 Orthodontics and Dentofacial Orthopaedics, Consultant Orthodontist, Hyderabad, IND; 2 Orthodontics and Dentofacial Orthopaedics, Practicing Orthodontist, Chhabria Dental Clinic, Thane, IND; 3 Orthodontics and Dentofacial Orthopaedics, Gian Sagar Dental College and Hospital, Jansla, IND; 4 Orthodontics and Dentofacial Orthopaedics, Specialist Orthodontist, Smile Dental Center, Kuwait, KWT; 5 Orthodontics and Dentofacial Orthopaedics, R. R. Dental College and Hospital, Udaipur, IND; 6 Orthodontics, The Oxford Dental College, Bangalore, IND; 7 Orthodontics, Sri Ramakrishna Dental College, Jaunpur, IND

**Keywords:** paediatric, mandibular positioning devices, rapid maxillary expansion, orthodontic treatment, obstructive sleep apnea

## Abstract

Obstructive sleep apnoea (OSA) poses a significant health burden globally, necessitating effective intervention strategies to mitigate its adverse consequences. Orthodontic treatment modalities offer promising avenues for addressing OSA by targeting the underlying anatomical abnormalities and restoring unobstructed airflow during sleep. This systematic search was conducted across multiple electronic databases using predefined search terms and inclusion criteria. Studies eligible for inclusion encompassed a range of study designs, including randomized controlled trials, prospective and retrospective studies, clinical trials, and observational studies. Outcome measures included changes in apnoea-hypopnoea index (AHI), oxygen saturation levels, polysomnographic variables, skeletal/cephalometric changes, nasal parameters, upper airway morphology, and clinical symptoms.

Initially, 756 records were identified through database searches, with 21 studies meeting the inclusion criteria after meticulous screening and selection. Orthodontic interventions, including rapid maxillary expansion (RME), personalized oral appliances, mandibular positioning devices, and comprehensive orthodontic protocols, demonstrated significant promise in ameliorating OSA symptoms among paediatric populations. Improvements in AHI, nasal resistance, sleep parameters, and upper airway dimensions were consistently observed across various studies, highlighting the potential of orthodontic treatments in mitigating OSA severity. This systematic review underscores the efficacy of orthodontic treatment modalities in addressing OSA among paediatric populations. Despite certain limitations in study design and outcome measures, the review emphasizes the need for further well-designed randomized controlled trials to validate and optimize these interventions for paediatric patients with OSA. Enhanced understanding and implementation of orthodontic treatments hold promise for alleviating the burden of OSA on global health and well-being.

## Introduction and background

Obstructive sleep apnoea (OSA) looms as a formidable threat to global public health, casting its pervasive shadow over millions worldwide [1]. Defined by recurrent episodes of upper airway obstruction during sleep, OSA disrupts the natural cycle of rest and rejuvenation, predisposing affected individuals to a cascade of adverse health consequences [1,2]. Among its repercussions, hypertension, cardiovascular disease, impaired cognitive function, and diminished quality of life stand prominent, underscoring the urgent need for efficacious intervention strategies [2]. In this context, orthodontic treatment modalities emerge as promising contenders in the ongoing battle against OSA, offering a diverse spectrum of approaches to address underlying anatomical aberrations and restore unobstructed airflow during sleep [3].

The landscape of orthodontic interventions for OSA is characterized by a mosaic of techniques and technologies, each imbued with unique nuances and considerations in its application [2,3]. Within this complex milieu, the pursuit of optimal therapeutic efficacy and patient-centric outcomes necessitates a comprehensive evaluation of existing modalities, guiding clinicians and researchers toward informed decision-making and evidence-based practice [3]. Within this framework, the present systematic review endeavours to illuminate the landscape of orthodontic treatment for OSA, synthesizing empirical evidence and clinical insights to inform best practices and foster advancements in the field.

At the crux of discussions surrounding orthodontic interventions for OSA lies the recognition of anatomical factors predisposing individuals to upper airway compromise during sleep [4]. From retrognathic mandibles to deficient maxillary dimensions and aberrant soft tissue dynamics, an array of structural anomalies may conspire to impede airflow and precipitate OSA [5]. In response, orthodontic modalities such as mandibular advancement devices (MADs) and maxillary expansion appliances (MEAs) have emerged as frontline interventions, leveraging the principles of craniofacial growth modification to alleviate airway constriction and mitigate OSA severity [6][7].

MADs, revered for their simplicity and non-invasiveness, exert their therapeutic effects by advancing the mandible relative to the maxilla, thereby enlarging the pharyngeal airspace and reducing the propensity for airway collapse [7]. Extensive documentation attests to their efficacy in improving polysomnographic parameters and ameliorating symptoms of OSA, positioning them as first-line options for mild to moderate cases and as adjunctive therapies in severe presentations [8]. Conversely, MEAs operate on the premise of maxillary expansion, employing controlled forces to augment maxillary dimensions and alleviate nasal obstruction, thus facilitating nasal breathing and mitigating OSA severity [9]. While their utility in paediatric populations and skeletally immature individuals is firmly established, the evidence supporting their efficacy in adult populations remains equivocal, necessitating further investigation and refinement [10].

Beyond conventional orthodontic appliances, emerging technologies such as temporary anchorage devices (TADs) and custom-designed mandibular advancement appliances herald new frontiers in OSA management, proffering precision-engineered solutions tailored to individualized anatomical considerations [11,12]. TADs, characterized by their biomechanical versatility and targeted application, afford precise control over dental and skeletal movements, facilitating the implementation of complex treatment protocols with heightened predictability and efficacy [11]. Similarly, custom-designed MADs, fabricated through computer-aided design and three-dimensional printing technologies, offer unparalleled customization and patient comfort, elevating the standard of care in OSA management to unprecedented heights.

However, amidst the burgeoning landscape of orthodontic treatment modalities for OSA, a paucity of high-quality evidence and standardized outcome measures persists, impeding the establishment of definitive treatment algorithms and guidelines [12,13]. By synthesizing existing evidence and delineating knowledge gaps, the present systematic review endeavours to chart a course towards enhanced clinical decision-making and improved patient outcomes in the realm of orthodontic treatment for OSA. This systematic review aims to evaluate the effectiveness of various orthodontic treatment modalities in managing OSA among paediatric populations, synthesizing empirical evidence and clinical insights to inform best practices and guide future research directions.

## Review

Materials and methodology

This systematic review meticulously adhered to the stringent guidelines delineated in the Preferred Reporting Items for Systematic Reviews and Meta-Analyses (PRISMA), thus ensuring a systematic and standardized approach to reporting. Designed to enhance the transparency and quality of systematic reviews, the PRISMA guidelines furnish a comprehensive checklist and flow diagram that reviewers are required to follow. This project was self-financed, and the protocol was registered at the PROSPERO site (registration number: CRD42024523135).apnoea-hypopnea index (AHI)

Before starting the review, we carefully developed a comprehensive methodology following the esteemed *Cochrane Handbook for Systematic Reviews of Interventions*. This handbook is highly regarded for its detailed guidance on every step of the systematic review process, from formulating research questions to selecting and evaluating studies, extracting data, and assessing bias. Our methodology included specific criteria for including and excluding studies, strategic methods for searching relevant databases, and detailed protocols for synthesizing and analysing data. We adopted this rigorous approach to minimize bias and ensure the reliability and validity of the review's findings.

Review Question

What are the outcomes of different orthodontic treatments for obstructive sleep apnoea (OSA)? It aims to analyse how effective various orthodontic interventions are in managing OSA, highlighting their pros and cons. By summarizing existing evidence, this review aims to guide clinical decisions and improve treatment approaches for OSA patients.

Search Strategy

When crafting a search strategy for the systematic review "Assessment of Orthodontic Treatments for Obstructive Sleep Apnoea," it was crucial to adopt a thorough and methodologically sound approach to find pertinent literature. This strategy targeted studies exploring orthodontic interventions for obstructive sleep apnoea (OSA), a condition marked by airway obstruction during sleep, causing breathing disruptions and insufficient oxygen levels.

To thoroughly gather relevant literature, electronic databases like PubMed, Embase, Cochrane Library, and Scopus were utilized, as they contained a wide range of peer-reviewed articles in orthodontics and sleep medicine. A combination of keywords was used to capture studies on orthodontic treatments and OSA, including terms like "Orthodontic treatment," "Obstructive sleep apnea," and "Continuous positive airway pressure (CPAP)." Boolean operators (AND, OR) helped construct search strings efficiently by combining these keywords. For instance, one search string was: ("Orthodontic treatment" OR "Orthodontic appliances") AND ("Obstructive sleep apnea" OR "OSA") AND ("Systematic review").

To refine the search results and ensure relevance, filters like publication date (e.g., last 20 years) and language (e.g., English) were applied. Manual screening of reference lists from relevant articles and systematic reviews complemented electronic searches, helping to identify additional studies. Grey literature sources, such as conference proceedings and clinical trial registries, were also searched for unpublished or ongoing research. Collaboration with experts and information specialists enhanced the search strategy. By doing so, the systematic review aimed to synthesize evidence on orthodontic treatments for OSA, providing insights for clinical practice and future research.

Inclusion and Exclusion Criteria

A variety of robust study designs, such as randomized controlled trials (RCTs), cohort studies, and observational studies, were included. The population study comprised paediatric patients diagnosed with OSA. Different orthodontic treatments for OSA were evaluated, including MADs, maxillomandibular advancement (MMA), orthognathic surgery, and orthodontic appliances. Studies comparing these treatments to each other, placebo, or no treatment were also considered. Outcome measures included changes in apnoea-hypopnoea index (AHI), oxygen saturation levels, quality of life indicators, and treatment adherence. Peer-reviewed articles, conference abstracts, and dissertations were included.

**Figure 1:**
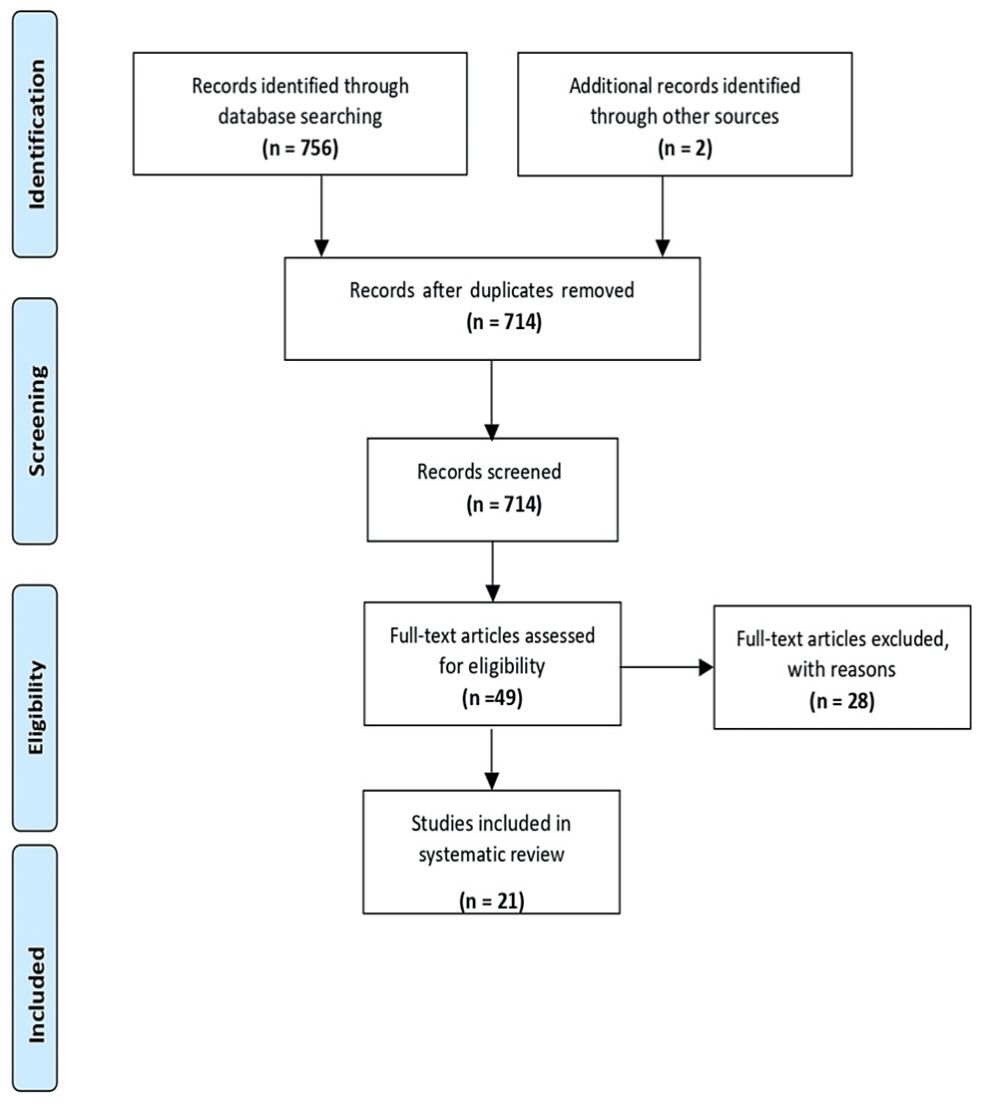
PRISMA flowchart for this systematic review PRISMA: Preferred Reporting Items for Systematic Reviews and Meta-Analyses.

Animal studies, case reports, editorials, letters, and reviews were excluded. Additionally, studies focusing only on adult populations or individuals with syndromic conditions were not considered. Investigations of treatments other than orthodontic interventions for OSA, such as continuous positive airway pressure (CPAP) therapy or surgical interventions not involving orthodontic techniques, were not included. Studies without a comparison group or with poorly controlled groups were also excluded. Any studies lacking relevant outcome measures for managing OSA were disregarded. Lastly, due to translation resource limitations, studies not in English were excluded. Adherence to PRISMA statement guidelines and a thorough search methodology enhanced the reliability and validity of this systematic review (Figure 1).

The determination of inclusion and exclusion criteria was guided by the aspects of Study design, Participants, Interventions, Comparisons, and Outcomes (SPICO) (Table 1).

**Table 1:** SPICO criteria for this systematic review SPICO: Study design, Participants, Interventions, Comparisons, and Outcomes; OSA: obstructive sleep apnoea; AHI: apnoea-hypopnoea index; SaO2: oxygen saturation

Criteria	Study details
Population	Study participants, aged 4 to 16 years, diagnosed with obstructive sleep apnoea (OSA), may present skeletal malocclusion and respiratory symptoms
Intervention	Orthodontic interventions include rapid maxillary expansion, rapid palatal expansion, personalized oral appliances, mandibular positioning devices, twin block appliances, and comprehensive orthodontic protocols
Comparison	Comparison between control and treatment group
Outcome	Key assessments include AHI, SaO_2_ levels, polysomnographic variables, skeletal/cephalometric changes, nasal parameters, upper airway morphology, and clinical symptoms
Study design	Prospective and retrospective studies, clinical trials, and observational studies

Screening and Selection

Two researchers collaborated on the search and screening process, achieving a substantial inter-rater agreement (κ = 0.83), ensuring reliability. The process involved four stages: Stage 1 excluded irrelevant citations, Stage 2 assessed titles and abstracts against inclusion criteria, and Stage 3 involved meticulous assessment by two independent reviewers, excluding studies with inappropriate designs or referencing deficiencies. Uncertainties were resolved through consensus or consultation with a second reviewer. In Stage 4, selected articles were subjected to a thorough examination and data extraction. Clinical methodologies and outcomes were critically appraised for reliability and relevance. This structured approach ensured methodological robustness and relevance of included articles. Adherence to high standards enhanced credibility and validity, reflected in the substantial κ coefficient.

Data Extraction

Initially, the primary author meticulously performed data extraction, ensuring accuracy. Subsequently, the second author reviewed and refined the data for completeness. Each eligible full-text article underwent independent data extraction, following a standardized format in Microsoft Excel 2013 (Microsoft, Redmond, WA, USA). Information was organized into sections including authorship, publication year, study design, participant demographics, intervention, comparator, and outcomes. This systematic approach facilitated clarity and analysis, ensuring all relevant details were captured accurately in Table 2.

**Table 2:** Data extraction sheet OSA: Obstructive sleep apnoea; AHI: apnoea-hypopnoea index; OHI: oral hygiene index; REM: rapid eye movement; NREM: non-rapid eye movement; RME: rapid maxillary expansion; ENT: ear, nose and throat; AI: apnoea index; PSG: polysomnography; RDI: respiratory disturbance index; SaO2: oxygen saturation; Epworth sleepiness scale (ESS); CT imaging: computed tomography imaging; NMSE: naso-maxillary skeletal expansion

Study	Population	Type of study	Mean age of the patients	Parameters checked	Intervention	Comparison	Outcome	Time period
Villa et al., 2007 [12]	Children aged 4-11 years with clinical signs of malocclusion, symptoms of OSA, and AHI >1	Prospective clinical trial	6.9 ± 2.2 years	Clinical score of OSA symptoms, AHI, OHI, arousal index, mean SaO_2_, REM, NREM, tonsillar hypertrophy, maxillary expansion	Orthodontic treatment with rapid maxillary expansion (RME)	Pre-treatment vs. post-treatment (baseline, 6 months, and 12 months)	Significant improvement in clinical score, AHI, OHI, and arousal index	November 2004 to April 2005
Pirelli et al., 2004 [13]	31 children with maxillary constriction, no adenotonsillar hypertrophy, BMI < 24 kg/m^2^, presence of OSAS, parents signed informed consent	Prospective trial	8.7 years (range: 6-12 years)	ENT evaluations, anterior rhinomanometry, nasal fibroscopy, panoramic radiographs, cephalometric radiographs, polysomnography, paediatric sleep questionnaire	Rapid maxillary expansion (RME)	Evaluations conducted at T0 (before orthodontic therapy), T1 (after 4 to 6 weeks with the device), T2 (4 months after end of treatment)	Significant improvement in nasal resistance, reduction in AHI, increased cross-sectional expansion of the maxilla, increased pyriform opening, improved sleep parameters	Initial examination, after four to six weeks with the device, and four months after the end of orthodontic treatment
Villa et al., 2002 [14]	32 children (20 males), age range: 4-10 years, mean age 7.1 ± 2.6 years	Randomized controlled study	7.1 ± 2.6 years (Overall), 6.86 ± 2.34 years (Intervention group), 7.34 ± 3.10 years (Control group)	Apnoea Index (AI), daytime symptoms like sleepiness, irritability, and tiredness; nighttime symptoms like habitual snoring, restless sleep, tonsillar hypertrophy, polysomnography	Personalized oral appliance for mandibular positioning, worn continuously except at mealtimes	Control group did not undergo therapy	Significant reduction in tonsillar hypertrophy in the treated group (66.7% vs. 14.3% in controls) and improvement in daytime and nighttime symptoms in the treated subjects. 64.2% of the treated subjects experienced a ≥50% reduction in AHI	Six-month trial period
Guilleminault et al., 2011 [15]	31 pre-pubertal children with OSA	Randomized controlled trial	6.5 ± 0.2 years	Clinical symptoms, polysomnography (PSG), apnoea-hypopnea index (AHI), respiratory disturbance index (RDI), lowest oxygen saturation (SaO_2_), tonsil and tongue position, nasal turbinates, nasal septum deviation, dental and orthodontic evaluations	Group 1: Adeno-tonsillectomy followed by orthodontics (rapid maxillary expansion) Group 2: Orthodontics (rapid maxillary expansion) followed by adeno-tonsillectomy	Pre-treatment vs. post-treatment 1 vs. post-treatment 2	Reduction in AHI and RDI, improvement in SaO_2_, clinical symptom improvement	Follow-up four weeks post-ENT surgery and three months post-orthodontic expansion
Marino et al., 2012 [16]	15 OSA syndrome children (eight boys and seven girls)	Longitudinal observational study	5.94 ± 1.64 years	Cephalometric variables (SNA, SNB, skeletal divergence, total facial height), respiratory disturbance index (RDI)	Rapid maxillary expansion (RME)	Baseline (T0) vs. post-treatment (T1)	Improvement in SNA and SNB angles in the improved (I) group compared to the stationary/worsened (SW) group	Mean follow-up period of 1.57 ± 0.58 years
Pirelli et al., 2012 [17]	80 children (43 boys and 37 girls) with OSAS, BMI <24 kg/m²	Longitudinal observational study	6–13 years (average 7.3 years)	Cephalometric variables, respiratory disturbance index, apnoea-hypoponea index (AHI), SpO_2_, polygraphic variables	Rapid maxillary expansion (RME) and adenotonsillectomy (AT)	Baseline (T0) vs. post-treatment (T1) and second phase evaluation (T2)	Improvements in cephalometric parameters, reduction in AHI, increase in maxillary width, normalization of SpO_2_	T1 - four months after treatment; T2 - after the completion of both RME and AT treatments, study conducted over eight years
Cozza et al., 2004 [18]	20 Caucasian children (10 boys and 10 girls) with OSA	Longitudinal observational study	Four to eight years (mean age 5.91)	Polysomnographic variables, obstructive apnoea-hypopnea index (AHI), minimum arterial oxygen saturation (min SaO_2_), arousal index, Epworth sleepiness scale (ESS)	Modified monobloc device worn nightly for six months	Baseline polysomnography vs. post-therapy polysomnography after six months	Decrease in median obstructive apnoea-hypopnoea index: from 7.88±1.81 episodes before treatment to 3.66±1.70 episodes after 6 months (p<0.001). ESS score reduced from 15.2±4.9 to 7.1±2.0. Arousal index and min SaO_2_ showed no significant change.	Six months
Caruso et al., 2023 [19]	14 paediatric patients (six males and eight females) aged between 6 and 10 years with mixed dentition and class III malocclusion associated with OSAS	Prospective cohort study	Median age: eight years	Skeletal variables, dental variables, upper airway space dimensions like nasopharynx, oropharynx, hypopharynx	Rapid maxillary expansion (RME) followed by Delaire mask treatment	Cephalometric variables measured before treatment (T0) and after treatment (T1)	Significant changes in dental variables, a skeletal variable (SNA), and upper airway space dimensions (nasopharynx and oropharynx) with p ≤ 0.05.	Orthodontic treatment duration: 18 months - RME activation: two rounds/day for 15 days - RME retained in mouth for 12 months
Pirelli et al., 2015 [20]	31 Caucasian children (19 boys)	Retrospective study	8.68 years	Clinical evaluation (Tanner stage, maxillary deficiency, cross-bites), otolaryngologic and orthodontic evaluation, PDSS, ESS, PSG, CT imaging	Rapid palatal expansion (RPE)	No control group specified	Significant reduction in AHI, improved oxygen saturation, stable anatomical changes, absence of OSA recurrence at final follow-up	12.3 ± 1.5 years (total), 12.0 ± 0.5 years (posttreatment follow-up)
Villa et al., 2015 [21]	Children aged 4-10 years referred to Paediatric Sleep Center, Sant’Andrea Hospital, Rome, Italy	Prospective longitudinal study	4-10 years	Clinical signs of malocclusion, tonsillar grading I-III, signs and symptoms of OSA, AHI > 1 as defined by PSG recording, parental written informed consent	Evaluation of RME	Baseline vs T1	Significant decrease in AHI (AHI T0: 4.7 ± 4.4 events/h vs AHI T1: 1.6 ± 1.4 events/h, p < 0.001)	Before and after treatment
Pirelli e al., 2019 [22]	Children presenting with snoring and clinical symptoms suggestive of abnormal breathing during sleep	Prospective observational study	10.5 years (range: 9–12 years)	PSG, CT imaging	Rapid maxillary expansion (RME) therapy	Pre-RME vs post-RME	Improvement in mid-palatal suture opening, maxillary width, nasal cavity width, first molar angulation, and pterygoid process distance	Pre-treatment (T0) and post-treatment (T1)
Pirelli et al., 2021 [23]	78 children with malocclusion	Prospective cohort study	8.5 years (range: 5-12 years)	Maxillary suture width, nasal width, molar angulation, pterygoid processes distance, nasal cavity dimensions, pharyngeal airway volume	Rapid maxillary expansion (RME)	Comparison between pre- and post-treatment measurements	Increase in mid-palatal suture opening, maxillary width, pterygoid processes distance, nasal cavity dimensions, and pharyngeal airway volume	Before orthodontic therapy (T0), after two months (T1) with device on, and four months after the end of orthodontic treatment (T2)
Kim et al., 2022 [24]	26 patients with OSA	Retrospective record-based study	13.6 ± 2.9 years (range: 9-18 years)	Transverse nasomaxillary dimensions, UA dimensions, HST parameters	Nasomaxillary skeletal expansion (NMSE)	Comparison between pre-treatment (T0) and post-treatment (T1) measurements	Significant expansion of nasal and upper airway dimensions, improved sleep parameters, and reduced symptoms	May 2016 to June 2019
Li et al., 2022 [25]	25 children aged 10-16 years who completed pre- and post-operative evaluations.	Observational study	10-16 years	Improvement in PSG metrics (AHI), clinical symptoms (OSA-18 scores), mid-palatal suture separation, nasal sidewall widening, dental expansion, nasal airflow pressure, and velocity	Treatment by transpalatal distraction (TPD) for nasomaxillary expansion	Comparison of pre- and post-operative data	Improvement in PSG metrics, clinical symptoms, successful mid-palatal suture separation, nasal sidewall widening, dental expansion, and reduced nasal airflow pressure and velocity	Until completion of orthodontic treatment
Chuang et al., 2019 [26]	Children suspected of pediatric OSA	Observational study	4-14 years	Clinical symptoms, AHI, RDI, BMI, age, sex, body weight, height, gestational age, birth body weight, OSA-18 scores, cephalometric data, upper airway morphology, PSG metrics, quality of life questionnaire	Treatment with a custom-designed oral appliance with built-in tongue bead (passive MFT) or no further treatment	Pre- and post-operative evaluations, PSG, lateral cephalometric X-ray	Significant improvement in PSG metrics, clinical symptoms, upper airway morphology, and quality of life survey scores; statistical analyses included Chi-square test, Mann-Whitney U test, and Wilcoxon signed-rank test	Before and after one year
Ghodke et al., 2014 [27]	Growing subjects with skeletal class II malocclusion	Prospective longitudinal study	8-14 years	Skeletal changes (SNA, SNB, FMA), PAP dimension changes (DOP, DHP, SPL, SPT, SPI), posterior pharyngeal wall thickness changes, age, sex, BMI, occlusion, crowding, rotations, follow-up duration	Correction with standard twin-block appliance, one-step mandibular advancement	Phase of pre-functional therapy with sectional fixed orthodontic appliance	Significant skeletal and PAP dimension changes. Maintenance of PPWT in treatment group. Different PPWT changes in control group	Before treatment (T0) and after approximately six months (T1)
Machado-Júnior et al., 2016 [28]	Children from Campinas at the physiological stage of mixed dentition	Prospective longitudinal study	Mean age: 8.13 years (experimental group) and 8.39 years (control group)	Clinical diagnosis of mandibular retrusion, symptoms of obstructive sleep apnoea (OSA), apnoea-hypopnoea index (AHI), sex distribution	Experimental subgroup: Mandibular advancement devices constructed based on neuro-occlusal rehabilitation principles and Pedro Planas' device, modified for the study	Control subgroup: No intraoral device or OSA treatment	Decrease in AHI in the experimental group, increase in the control group	Initial examination and after 12 months
Concepción Medina et al., 2022 [29]	39 children: 20 in activator group, 19 control	Prospective longitudinal study	10.9±0.9 years (activator group), 9.8±1.4 years (control group)	Skeletal pattern, SNA angle, SNB angle, ANB angle, BMI, sleep-related breathing disorder symptoms, upper airway linear width, cephalometric measurements, at-home sleep-breathing monitoring indicators	Activator group: Wore Andresen functional activator appliance	Control group: No activator appliance	Improved sleep breathing patterns, widened upper airway, decreased severity of sleep breathing disturbances	Initial assessment and after functional therapy
Zhang et al., 2013 [30]	46 patients from the Department of Orthodontics, Wuhan University	Prospective longitudinal study	9.7±1.5 years	Cervical vertebrae maturation indices, mandibular retrognathia (ANB, SNB, incisor overjet), snoring habit, OSA (AHI), BMI	Customized twin block appliances	Pre-treatment vs post-treatment	Improved airway, reduced AHI, increased lowest SaO_2_, No significant change in mean SaO_2_, forward movement of mandible, improved facial convexity	Before and after TB treatment
Zhao et al., 2018 [31]	Patients aged 12-14 years from the Department of Orthodontics, Wuhan University	Retrospective comparative study	12.3 ± 1.2 years	Full permanent dentition, distal molar relationship (ANB ≥ 4), hyperdivergent skeletal growth (SNGoMe ≥ 36), crowding ≤ 3 mm, PSG findings, lateral cephalometric radiograph	Comprehensive orthodontic treatment protocol by same orthodontist	OSAHS group vs control group	Improved craniofacial structures, changes in cephalometric measurements, normalized overbite and overjet	Before and after treatment
Zreaqat et al., 2023 [32]	34 polysomnography - proven OSA children with class II mandibular retrognathic skeletal malocclusion	Interventional	8-12 years	Upper airway parameters/dimensions, apnoea-hypopnoea indexes (AHIs)	Myofunctional twin-block therapy	Treatment vs control group	Increase in upper airway volume, increase in minimal cross-sectional area (MCA), decrease in AHI	Pre- and posttreatment

Assessment of Risk of Bias

The assessment of bias in this systematic review employed the ROBINS-I (Risk Of Bias In Non-randomised Studies - of Interventions) tool, designed for non-randomized intervention studies. Each study was subjected to meticulous evaluation across several domains. Firstly, confounding bias was scrutinized to ascertain whether relevant confounders were adequately addressed and adjusted for. Studies lacking such adjustments were deemed to have a higher risk of bias. Secondly, selection bias was assessed by examining participant recruitment methods to ensure comparability between groups. Studies with flawed selection processes were flagged for higher bias (Table 3).

**Table 3:** Assessment of the risk of bias

Author	Bias due to confounding	Bias in the selection of participants in the study	Bias in the classification of interventions	Bias due to deviations from intended interventions	Bias due to missing data	Bias in the measurement of outcomes	Bias in the selection of the reported result	Overall bias
Villa et al., 2007 [12]	2	2	3	3	2	2	2	16
Pirelli et al., 2004 [13]	2	2	1	2	1	2	2	12
Villa et al., 2002 [14]	2	3	2	2	1	2	2	14
Guilleminault et al., 2011 [15]	2	3	1	2	3	3	1	14
Marino et al., 2012 [16]	2	3	2	2	2	1	2	14
Pirelli et al., 2012 [17]	2	2	1	1	3	1	2	10
Cozza et al., 2004 [18]	2	2	3	2	1	2	2	14
Caruso et al., 2023 [19]	2	2	3	3	2	2	2	16
Pirelli e al., 2015 [20]	2	3	1	2	2	3	1	13
Villa Met al., 2015 [21]	2	3	1	1	2	1	1	11
Pirelli et al., 2019 [22]	2	3	1	2	3	3	1	14
Pirelli et al., 2021 [23]	2	2	1	1	3	1	2	10
Kim et al., 2022 [24]	2	2	3	2	1	2	2	14
Li et al., 2022 [25]	2	2	3	3	2	2	2	16
Chuang et al., 2019 [26]	2	3	1	2	2	3	1	13
Ghodke et al., 2014 [27]	2	3	1	1	2	1	1	11
Machado-Júnior et al., 2016 [28]	2	2	3	3	2	2	2	16
Concepción Medina et al., 2022 [29]	2	2	1	2	1	2	2	12
Zhang et al., 2013 [30]	2	3	2	2	1	2	2	14
Zhao et al., 2018 [31]	2	3	1	2	3	3	1	14
Zreaqat et al., 2023 [32]	2	3	2	2	2	1	2	14

Intervention classification bias was also scrutinized to ensure consistent and accurate intervention categorization. Deviations from intended interventions were analysed to assess participant adherence and management of deviations. Studies with uncontrolled deviations were deemed to have higher bias. Missing data bias was another focal point, emphasizing how missing data were addressed and their impact on results. Inadequate handling of missing data raised concerns about bias. Additionally, outcome measurement bias was evaluated based on measurement method reliability and consistency. Inconsistent or unreliable measurements increased the risk of bias. Lastly, outcome reporting bias was examined to identify selective outcome reporting. Studies failing to report all prespecified outcomes or showing selective reporting were considered to have a higher risk of bias. The overall bias risk for each study was synthesized from these assessments, offering a comprehensive evaluation of methodological rigour and potential biases. The structured approach provided insights into the reliability and validity of the reviewed studies, enhancing the credibility of the systematic review's findings.

Results

Initially, we found 756 records through database searches, plus two more from alternative sources, totalling 758. After removing duplicates, we had 714 unique records. Screening led to assessing 49 records for eligibility, with 665 exclusions based on preset criteria. We then scrutinized full-text articles, excluding 28 studies for various reasons. Ultimately, 21 studies met our inclusion criteria and were included in the systematic review. This methodical process allowed for a thorough examination of the literature, enabling a comprehensive synthesis of evidence relevant to our research question.

The systematic review on the evaluation of orthodontic treatment modalities for OSA encompasses a comprehensive analysis of various studies focused on addressing this prevalent sleep disorder in paediatric populations. Across the diverse studies reviewed, orthodontic interventions showed significant promise in ameliorating symptoms associated with OSA among children. Villa et al. (2007) conducted a prospective clinical trial targeting children aged 4-11 years exhibiting clinical signs of malocclusion and symptoms of OSA. Their findings demonstrated a marked improvement in clinical score of OSA symptoms, AHI, oral hygiene index (OHI), arousal index, mean arterial oxygen saturation (SaO_2_), and various other parameters following orthodontic treatment with rapid maxillary expansion (RME) [12].

Similarly, Pirelli et al. (2004) and Villa et al. (2002) underscored the efficacy of rapid maxillary expansion (RME) in addressing OSA-related concerns among paediatric patients, with notable improvements observed in nasal resistance, reduction in AHI, and enhancement of sleep parameters [13,14]. Further corroborating these findings, studies by Cozza et al. (2004) and Caruso et al. (2023) highlighted significant reductions in obstructive AHI and improvements in sleep quality following orthodontic interventions, emphasizing the potential of such modalities in mitigating OSA symptoms [18,19].

Moreover, longitudinal studies by Pirelli et al. (2012) and Zhao et al. (2018) showcased sustained improvements in cephalometric parameters, reduction in AHI, and normalization of oxygen saturation levels post-orthodontic treatment, suggesting the enduring efficacy of these interventions in managing OSA [17,31]. Additionally, Chuang et al. (2019) and Zreaqat et al. (2023) provided further insights into the benefits of orthodontic therapies, including nasomaxillary skeletal expansion (NMSE) and myofunctional twin-block therapy, in enhancing upper airway dimensions and reducing AHI among paediatric OSA patients [26,32].

Discussion

The systematic review provides a comprehensive analysis of various orthodontic interventions aimed at managing OSA in children. The discussion section synthesizes the findings from multiple studies to elucidate the efficacy and impact of different orthodontic approaches in addressing OSA symptoms and improving patients' quality of life.

In a prospective clinical trial by Villa et al. (2007), children with malocclusion and symptoms of OSA underwent orthodontic treatment with rapid maxillary expansion (RME) [12]. The study observed significant improvements in OSA symptoms, AHI, and other relevant parameters, indicating the potential of RME in alleviating OSA in paediatric patients. Similarly, Pirelli et al. (2004) investigated the effects of RME in children with maxillary constriction and OSA [13]. Their prospective trial demonstrated notable enhancements in nasal resistance, reduction in AHI, and improvements in sleep parameters post-treatment, highlighting the favourable outcomes associated with RME in managing OSA.

In a randomized controlled study led by Villa et al. (2002), personalized oral appliances for mandibular positioning showed promising results in reducing tonsillar hypertrophy and relieving daytime and nighttime symptoms in children with OSA [14]. This underscores the effectiveness of personalized oral appliances as a therapeutic option for paediatric OSA. Guilleminault et al. (2011) compared the outcomes of adeno-tonsillectomy followed by orthodontics versus orthodontics followed by adeno-tonsillectomy in pre-pubertal children with OSA [15]. Both groups experienced improvements in AHI and respiratory disturbance index (RDI), highlighting the significance of multimodal approaches in managing paediatric OSA.

Marino et al. (2012) conducted a longitudinal observational study evaluating the impact of RME on cephalometric variables and respiratory disturbance index in children with OSA. They observed significant improvements in skeletal angles post-treatment, indicating the effectiveness of RME in correcting anatomical abnormalities associated with OSA [16]. Furthermore, studies by Pirelli et al. (2012, 2019, 2021) and Caruso et al. (2023) investigated the efficacy of RME in improving maxillary dimensions, reducing AHI, and enhancing upper airway parameters in paediatric OSA patients, corroborating the favourable outcomes of orthodontic interventions in OSA management [17,19,22,23].

Furthermore, retrospective studies conducted by Pirelli et al. (2015) and Kim et al. (2022) demonstrated sustained improvements in AHI, oxygen saturation, and anatomical changes following RME, reinforcing the long-term efficacy of orthodontic treatments in paediatric OSA [20,24]. Additionally, investigations by Li et al. (2022) and Chuang et al. (2019) examined the effectiveness of trans palatal distraction and custom-designed oral appliances in enhancing polysomnography (PSG) metrics, alleviating clinical symptoms, and improving upper airway morphology in paediatric OSA patients [25,26]. These studies offer valuable insights into alternative orthodontic modalities for managing OSA in children.

While the systematic review on orthodontic treatment for paediatric OSA offers valuable insights, it's important to acknowledge its limitations. The included studies varied in design, sample size, and follow-up duration, potentially introducing heterogeneity and limiting generalizability. Most studies relied on subjective measures, which could be prone to observer bias and may not fully capture OSA's multifactorial nature. Additionally, many studies were observational or retrospective, lacking rigorous control groups or randomization, thus limiting causal inferences. The long-term sustainability of orthodontic interventions remains unclear due to short follow-up periods in some studies. While the review covered various orthodontic modalities, including RME and personalized oral appliances, further research, especially well-designed randomized controlled trials, is needed to compare their effectiveness, optimal timing, and duration. Despite these limitations, the review highlights the potential of orthodontic treatments in addressing paediatric OSA, emphasizing the need for future research to fill existing gaps and strengthen the evidence base in this area.

## Conclusions

In summary, this systematic review thoroughly evaluates various orthodontic treatments for pediatric obstructive sleep apnoea (OSA). The analysis highlights the effectiveness of interventions like rapid maxillary expansion (RME), personalized oral appliances, and mandibular positioning devices in alleviating OSA symptoms and enhancing clinical outcomes in children. Although some limitations exist in study design and outcome measures, the review underscores the promising role of orthodontic treatments in managing OSA in children. Further well-designed randomized controlled trials are needed to confirm and refine these interventions for pediatric OSA patients.

## References

[1] Marcus CL, Brooks LJ, Draper KA (2012). Diagnosis and management of childhood obstructive sleep apnea syndrome. Pediatrics.

[2] Katz ES, D'Ambrosio CM (2010). Pediatric obstructive sleep apnea syndrome. Clin Chest Med.

[3] Alexander NS, Schroeder JW Jr (2013). Pediatric obstructive sleep apnea syndrome. Pediatr Clin North Am.

[4] Schwengel DA, Dalesio NM, Stierer TL (2014). Pediatric obstructive sleep apnea. Anesthesiol Clin.

[5] Arens R, Muzumdar H (2010). Childhood obesity and obstructive sleep apnea syndrome. J Appl Physiol (1985).

[6] Alonso-Álvarez ML, Cordero-Guevara JA, Terán-Santos J (2014). Obstructive sleep apnea in obese community-dwelling children: the NANOS study. Sleep.

[7] Kohler MJ, Thormaehlen S, Kennedy JD (2009). Differences in the association between obesity and obstructive sleep apnea among children and adolescents. J Clin Sleep Med.

[8] Xanthopoulos MS, Gallagher PR, Berkowitz RI, Radcliffe J, Bradford R, Marcus CL (2015). Neurobehavioral functioning in adolescents with and without obesity and obstructive sleep apnea. Sleep.

[9] Horwood L, Brouillette RT, McGregor CD, Manoukian JJ, Constantin E (2014). Testing for pediatric obstructive sleep apnea when health care resources are rationed. JAMA Otolaryngol Head Neck Surg.

[10] Nixon GM, Kermack AS, Davis GM, Manoukian JJ, Brown KA, Brouillette RT (2004). Planning adenotonsillectomy in children with obstructive sleep apnea: the role of overnight oximetry. Pediatrics.

[11] Tan HL, Gozal D, Ramirez HM, Bandla HP, Kheirandish-Gozal L (2014). Overnight polysomnography versus respiratory polygraphy in the diagnosis of pediatric obstructive sleep apnea. Sleep.

[12] Villa MP, Malagola C, Pagani J, Montesano M, Rizzoli A, Guilleminault C, Ronchetti R (2007). Rapid maxillary expansion in children with obstructive sleep apnea syndrome: 12-month follow-up. Sleep Med.

[13] Pirelli P, Saponara M, Guilleminault C (2004). Rapid maxillary expansion in children with obstructive sleep apnea syndrome. Sleep.

[14] Villa MP, Bernkopf E, Pagani J, Broia V, Montesano M, Ronchetti R (2002). Randomized controlled study of an oral jaw-positioning appliance for the treatment of obstructive sleep apnea in children with malocclusion. Am J Respir Crit Care Med.

[15] Guilleminault C, Monteyrol PJ, Huynh NT, Pirelli P, Quo S, Li K (2011). Adeno-tonsillectomy and rapid maxillary distraction in pre-pubertal children, a pilot study. Sleep Breath.

[16] Marino A, Ranieri R, Chiarotti F, Villa MP, Malagola C (2012). Rapid maxillary expansion in children with obstructive sleep apnoea syndrome (OSAS). Eur J Paediatr Dent.

[17] Pirelli P, Saponara M, Guilleminault C (2012). Rapid maxillary expansion before and after adenotonsillectomy in children with obstructive sleep apnea. Somnologie.

[18] Cozza P, Gatto R, Ballanti F, Prete L (2004). Management of obstructive sleep apnoea in children with modified monobloc appliances. Eur J Paediatr Dent.

[19] Caruso S, Lisciotto E, Caruso S (2023). Effects of rapid maxillary expander and Delaire mask treatment on airway sagittal dimensions in pediatric patients affected by class III malocclusion and obstructive sleep apnea syndrome. Life (Basel).

[20] Pirelli P, Saponara M, Guilleminault C (2015). Rapid maxillary expansion (RME) for pediatric obstructive sleep apnea: a 12-year follow-up. Sleep Med.

[21] Villa MP, Rizzoli A, Rabasco J (2015). Rapid maxillary expansion outcomes in treatment of obstructive sleep apnea in children. Sleep Med.

[22] Pirelli P, Fanucci E, Giancotti A, Di Girolamo M, Guilleminault C (2019). Skeletal changes after rapid maxillary expansion in children with obstructive sleep apnea evaluated by low-dose multi-slice computed tomography. Sleep Med.

[23] Pirelli P, Fiaschetti V, Fanucci E, Giancotti A, Condo' R, Saccomanno S, Mampieri G (2021). Cone beam CT evaluation of skeletal and nasomaxillary complex volume changes after rapid maxillary expansion in OSA children. Sleep Med.

[24] Kim JE, Hwang KJ, Kim SW, Liu SY, Kim SJ (2022). Correlation between craniofacial changes and respiratory improvement after nasomaxillary skeletal expansion in pediatric obstructive sleep apnea patients. Sleep Breath.

[25] Li K, Iwasaki T, Quo S, Li C, Young K, Leary E, Guilleminault C (2022). Persistent pediatric obstructive sleep apnea treated with skeletally anchored transpalatal distraction. Orthod Fr.

[26] Chuang LC, Hwang YJ, Lian YC, Hervy-Auboiron M, Pirelli P, Huang YS, Guilleminault C (2019). Changes in craniofacial and airway morphology as well as quality of life after passive myofunctional therapy in children with obstructive sleep apnea: a comparative cohort study. Sleep Breath.

[27] Ghodke S, Utreja AK, Singh SP, Jena AK (2014). Effects of twin-block appliance on the anatomy of pharyngeal airway passage (PAP) in class II malocclusion subjects. Prog Orthod.

[28] Machado-Júnior AJ, Signorelli LG, Zancanella E, Crespo AN (2016). Randomized controlled study of a mandibular advancement appliance for the treatment of obstructive sleep apnea in children: A pilot study. Med Oral Patol Oral Cir Bucal.

[29] Concepción Medina C, Ueda H, Iwai K, Kunimatsu R, Tanimoto K (2022). Changes in airway patency and sleep-breathing in healthy skeletal Class II children undergoing functional activator therapy. Eur Oral Res.

[30] Zhang C, He H, Ngan P (2013). Effects of twin block appliance on obstructive sleep apnea in children: a preliminary study. Sleep Breath.

[31] Zhao T, Ngan P, Hua F (2018). Impact of pediatric obstructive sleep apnea on the development of class II hyperdivergent patients receiving orthodontic treatment: a pilot study. Angle Orthod.

[32] Zreaqat M, Hassan R, Samsudin AR, Alforaidi S (2023). Effects of twin-block appliance on upper airway parameters in OSA children with class II malocclusion and mandibular retrognathia: a CBCT study. Eur J Pediatr.

